# Serum Magnesium and Ventricular Repolarization Heterogeneity: A Case–Control Study Based on Electrocardiographic Parameters

**DOI:** 10.1007/s12011-026-05028-2

**Published:** 2026-02-17

**Authors:** Ramazan Aslan, Halil İbrahim Özkan, Faik Özel, Murat Özmen, İsa Ardahanlı

**Affiliations:** 1 Department of Cardiology, Bilecik Training and Research Hospital, Bilecik, Türkiye; 2 Department of Medical Biochemistry, Balıkesir University Faculty of Medicine, Balıkesir, Türkiye; 3 Department of Internal Medicine, Bilecik Training and Research Hospital, Bilecik, Türkiye; 4 Department of Cardiology, Health Sciences University Erzurum Faculty of Medicine, Erzurum, Türkiye; 5 Department of Cardiology, School of Medicine, Seyh Edebali University, Bilecik, 11000 Turkey

**Keywords:** Hypomagnesemia, Ventricular repolarization, Tp-e, QTc, Arrhythmia risk, ECG

## Abstract

This study investigated the changes in ventricular repolarization parameters assessed by electrocardiography (ECG) in the presence of hypomagnesemia and the relationship between these parameters and serum magnesium levels. A total of 160 individuals were included in this retrospective, observational study, who were divided into two groups according to serum magnesium levels: hypomagnesemia group (*n* = 80; serum Mg < 1.7 mg/dL) and normomagnesemia control group (*n* = 80; serum Mg 1.8–2.2 mg/dL). The two groups were matched for age and gender. Ventricular repolarization parameters such as QT, corrected QT (QTc), T peak-to-T end interval (Tp-e), Tp-e/QT, Tp-e/QTc, QRS duration, and heart rate were measured from the ECG recordings of all participants and compared. In the hypomagnesemia group, QT, QTc, Tp-e, Tp-e/QT, and Tp-e/QTc parameters were significantly prolonged compared to the control group (for all *p* < 0.001). A significant negative correlation was found between serum magnesium level and QTc (*r* = − 0.437), Tp-e (*r* = − 0.457), Tp-e/QT (*r* = − 0.288), and Tp-e/QTc (*r* = − 0.281). In multivariate regression analyses, magnesium level was determined as an independent predictor for both QTc duration (β = − 20.3, *p* < 0.001) and Tp-e/QTc ratio (β = − 0.048, *p* = 0.001), despite controlling for potential confounding variables. The findings show that serum magnesium level is independent and clinically significant on ventricular repolarization duration and heterogeneity. Hypomagnesemia was associated with alterations in ventricular repolarization parameters, reflecting increased repolarization heterogeneity. Our findings suggest that low serum magnesium is independently associated with prolonged Tp-e/QTc and QTc intervals, highlighting its role in ventricular repolarization heterogeneity.

## Introduction

 Magnesium (Mg²⁺) is the second most abundant intracellular cation in the human body after potassium. It plays a critical role in regulating biochemical processes as a cofactor of more than 300 enzymes [1–3]. It is an indispensable element for the healthy functioning of cellular energy metabolism, DNA and RNA synthesis, oxidative phosphorylation, neuromuscular transmission, muscle contraction, and ion transport mechanisms [4, 5]. Beyond all these processes, magnesium ions have a direct, decisive effect on the electrical and mechanical stability of the cardiovascular system. In particular, by regulating the transmembrane movements of sodium (Na⁺), potassium (K⁺), and calcium (Ca²⁺) ions, it maintains cardiomyocyte membrane potential in balance, modulates the duration of the action potential, and thus helps prevent the development of arrhythmias [6, 7].

Hypomagnesemia, i.e., serum magnesium levels falling below the reference range (< 1.7 mg/dL), is an electrolyte disorder often overlooked in clinical practice but can have potentially serious consequences. Hypomagnesemia is often associated with clinical conditions such as diabetes mellitus, gastrointestinal losses, alcohol abuse, diuretic use, malnutrition, and chronic kidney disease. Low serum magnesium levels can cause ion imbalances and cell membrane instability in cardiac muscle cells, prolonging repolarization time and increasing the risk of developing potentially life-threatening ventricular arrhythmias such as torsades de pointes [8]. The ventricular repolarization process that occurs in the 3rd phase of the cardiac action potential represents the preparation of the myocardium for the next stimulus. This physiological event can be indirectly assessed by surface electrocardiography (ECG). Although QT and corrected QT (QTc) intervals have been accepted as classical indicators of repolarization duration for many years, there are criticisms that these parameters do not adequately reflect transmural repolarization heterogeneity. In recent years, the Tp-e interval, which is the distance between the peak and the endpoint of the T wave, and the Tp-e/QT and Tp-e/QTc ratios obtained by dividing it by QT or QTc have come to the forefront as more sensitive indicators of transmural repolarization dispersion [9, 10]. Tp-e and its derivative ratios have become quantitative indicators of increased transmural dispersion, reflecting differences in repolarization time between endocardial, epicardial, and M-cells [11]. Increases in these parameters have been directly associated with increased arrhythmogenic potential and considered important predictors of malignant ventricular arrhythmias, especially torsades de pointes [12]. Therefore, the use of these electrocardiographic indicators is gaining increasing importance not only in academic research but also in clinical decision support systems.

Many studies on the electrophysiological effects of magnesium are based on animal models and cell culture experiments. However, clinical studies systematically examining new-generation ECG markers (Tp-e, Tp-e/QT, Tp-e/QTc) affecting ventricular repolarization in isolated hypomagnesemia in humans are limited. In most of the existing studies in the literature, patient populations are heterogeneous, and important confounding factors such as concomitant potassium or calcium imbalances, structural heart diseases, or the use of medications that may affect the QT interval are not adequately controlled.

The primary purpose of this study is to evaluate the changes in repolarization markers such as QT, QTc, Tp-e, Tp-e/QT, and Tp-e/QTc in individuals diagnosed with isolated hypomagnesemia by comparing them with a healthy control group matched for age and gender. In addition, by ensuring homogeneity in potential confounding variables such as body mass index and potassium and calcium levels between the groups, the electrophysiological effects of magnesium levels were tried to be revealed more clearly. The data to be obtained to provide strong evidence for the usability of new generation ECG-based parameters in the early prediction of arrhythmic risks due to hypomagnesemia and to guide clinical practices.

## Materials and Methods

### Study Design and Population

This case-control retrospective study was conducted among adult individuals who applied to the cardiology outpatient clinic of Bilecik Education and Research Hospital between January 2025 and March 2025. The study’s target population consisted of individuals who applied for cardiovascular evaluation and had complete laboratory data regarding electrolyte levels. The study population was retrospectively screened from the hospital’s digital patient record system, and appropriate individuals were selected according to the inclusion-exclusion criteria. Participants were divided into two groups according to the detected serum magnesium levels. The hypomagnesemia group included individuals with serum magnesium levels < 1.7 mg/dL (*n* = 80). The normomagnesemia control group consisted of individuals with serum magnesium levels between 1.8 and 2.2 mg/dL and without any electrolyte imbalance or structural heart disease (*n* = 80). The control group was selected by matching individuals in the hypomagnesemia group at a 1:1 ratio of age and gender. Thus, the potential effects of age and gender on repolarization parameters were minimized. This configuration aimed to evaluate the effect of serum magnesium levels on ventricular repolarization parameters in a more isolated and controlled manner. This matching was performed using a frequency-matching approach rather than individual pair matching; therefore, independent-sample statistical tests were applied for between-group comparisons.

Patients were retrospectively and consecutively screened from the institutional electronic medical record system during the study period. Of the initially evaluated individuals, patients were excluded due to electrolyte imbalances other than magnesium, use of QT-prolonging medications, known structural heart disease, renal dysfunction (GFR < 60 mL/min/1.73 m²), anemia, acute infection, poor-quality ECG recordings, or incomplete laboratory data. Individuals with serum magnesium levels between 1.7 and 1.8 mg/dL were excluded to ensure clear separation between study groups. Exclusion criteria include electrolyte imbalances (such as hypokalemia, hypercalcemia, etc.), the use of medications that affect the QT interval (e.g., antiarrhythmics, antidepressants), known structural heart disease (cardiomyopathy, ischemic heart disease), ECG abnormalities including bundle branch block (LBBB, RBBB) or preexcitation syndrome, acute infection or sepsis, renal failure (defined as GFR < 60 mL/min/1.73 m²), and anemia (Hb < 11 g/dL) or hematological diseases.

### Data Collection and Laboratory Measurements

Demographic, clinical, and laboratory data of the participants included in the study were obtained retrospectively through the electronic health record system. Information on the participants’ age, gender, height, weight, body mass index (BMI), medical history, and regularly used medications were scanned through patient files and outpatient clinic follow-up notes. Biochemical and hematological parameters were analyzed in the hospital laboratory using international quality standards from fasting blood samples taken at the time of admission to the hospital. Serum magnesium, potassium, sodium, calcium, creatinine, and urea levels were measured on fully automatic biochemistry analyzers; hemoglobin and hematocrit values ​​were measured on hematological analyzers. All measurements were performed under the same laboratory conditions using the method and reference ranges recommended by the manufacturer. These data constituted the basic data set of the study in terms of evaluating possible biochemical confounding factors and ensuring homogeneity between the groups.

### Electrocardiographic Evaluation

**Fig. 1:**
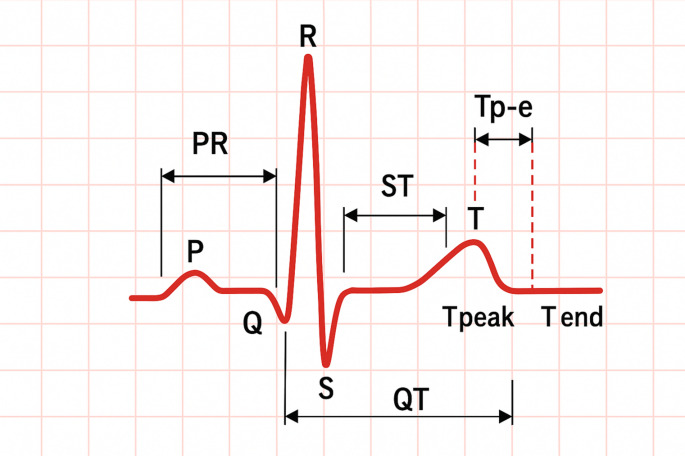
Ventricular repolarization parameters on ECG

Surface electrocardiography was used to evaluate ventricular repolarization parameters relevant to arrhythmogenic risk. QT and corrected QT (QTc) intervals were assessed as conventional markers of repolarization duration, while Tp-e interval and Tp-e/QT and Tp-e/QTc ratios were evaluated as indices of transmural repolarization dispersion. These waves and intervals provide clinically critical information in the evaluation of the electrical and rhythmic functionality of the heart (Fig. 1).

All participants underwent standard 12-lead surface electrocardiography (ECG) at rest and in the supine position. ECG recordings were obtained with the Nihon Kohden Cardiofax C ECG-2350 device using a paper speed of 25 mm/sec and a calibration standard of 10 mm/mV. All measurements were performed under the same device and technical conditions, thus minimizing technical variation. Electrocardiographic parameters were evaluated using manual and digital magnification methods on ECG outputs. Measurements were taken on the most appropriate three consecutive cardiac cycles for each individual, and the average of these values ​​was calculated. QT, QTc, and Tp-e measurements were primarily obtained from lead V5. If lead V5 was unsuitable due to low T-wave amplitude or artifacts, lead V6 was used, followed by lead V4 according to a predefined hierarchy. The end of the T wave was determined using the tangent method, defined as the intersection of the tangent to the terminal downslope of the T wave with the isoelectric baseline. Lead V5 was used in approximately 70% of measurements, followed by V6 in 20% and V4 in 10% of cases. The measurements of the parameters were performed independently by two experienced cardiologists. In cases where significant differences were detected between the measurements, a consensus was reached by obtaining the opinion of a third senior cardiologist. A third cardiologist was required for adjudication in fewer than 10% of ECG measurements. Interobserver variability was assessed in a randomly selected subset of 15 ECG recordings. The intraclass correlation coefficient (ICC) demonstrated excellent agreement for QTc (ICC = 0.91) and Tp-e measurements (ICC = 0.89). This approach was implemented to minimize interobserver variation and increase the reliability of the measurements [13, 14].

The main electrocardiographic parameters evaluated are as follows:

- QT interval: The time from the beginning of the QRS complex to the end of the T wave was determined by taking the average of three separate complexes.
- QTc interval: The QT duration corrected by heart rate was calculated using the Bazett formula (QTc = QT/√RR).
- Tp-e interval: The time between the peak and the end of the T wave; usually measured in leads V5 or V6, or in leads V4 or V2 depending on technical suitability [15, 16].
- Tp-e/QT and Tp-e/QTc ratios: Calculated by taking the ratio of the Tp-e interval to the QT and QTc durations, respectively, and evaluated as an indicator of transmural repolarization dispersion.
- QRS duration: The time from the beginning to the end of the complex was measured in milliseconds.
- Heart rate (HR): The average heart rate was calculated over the RR intervals.

ECG recordings with poor image quality, artifacts, or technically challenging to interpret were excluded from the analysis. This systematic and multi-layered measurement approach achieved high measurement accuracy and methodological consistency in the evaluation of ventricular repolarization parameters.

### Echocardiographic Evaluation

All participants underwent transthoracic echocardiography (TTE) at rest and in the left lateral decubitus position. The evaluations were performed by an experienced cardiologist using a Vivid S60N (GE Healthcare, Chicago, IL, USA) brand high-resolution ultrasound device in the hospital echocardiography laboratory. Standard parasternal long-axis, short-axis, and apical four-chamber viewing windows were used during the imaging. Left ventricular ejection fraction (LVEF) was calculated using the modified Simpson method from two-dimensional images. Left atrial diameter (LA) was evaluated from the parasternal long-axis window, and interventricular septum thickness (IVS) was evaluated using end-diastolic measurement with the M-mode imaging technique. All measurements were performed according to the current guidelines published by the American Society of Echocardiography (ASE) and the European Association of Cardiovascular Imaging (EACVI) [17]. The average of measurements obtained from at least three separate cardiac cycles was taken for each parameter, and artifact-containing or technically inadequate images were excluded from the analysis. This method aimed to evaluate structural cardiac features reliably and objectively. Echocardiographic data were analyzed with electrocardiographic parameters, and the effects of serum magnesium levels on cardiac functions were comprehensively investigated.

### Statistical Analysis

All statistical analyses were performed using IBM SPSS Statistics version 26.0 (IBM Corp., Armonk, NY, USA) and R Studio software (v4.2.2). The Shapiro-Wilk test tested the assumption of normal distribution for continuous variables. When necessary, Skewness and kurtosis coefficients were examined and supported with visual analysis (histogram, Q-Q plot). Variance homogeneity was assessed using the Levene test. Normally distributed data were presented as mean ± standard deviation (SD), and data not showing normal distribution were presented as median (min-max). Categorical variables were expressed as numbers and percentages (%). In pairwise group comparisons, appropriate statistical tests were used according to the distribution properties of the data. An independent sample t-test was preferred for analyzing continuous variables showing normal distribution. A non-parametric Mann–Whitney U test was applied for continuous variables that did not show normal distribution. Pearson chi-square test was used to compare categorical variables; Fisher’s exact test was used in cases where cell frequencies were insufficient. All statistical tests were based on two-tailed hypotheses, and *p* < 0.05 was accepted as the statistical significance limit. These analyses were performed to obtain reliable and valid results in evaluating differences between the study groups.

The study performed effect size analyses to evaluate the clinical equivalent of statistical significance related to pairwise group comparisons. Cohen’s d coefficient was calculated for the independent sample t-test results applied within the scope of parametric tests, and the r value was used as the effect size coefficient for variables applied to the nonparametric Mann–Whitney U test. The obtained effect size values ​​were interpreted by the threshold values ​​accepted in the literature (0.2: small, 0.5: medium, 0.8: significant effect). This evaluation was aimed to provide a more holistic perspective on whether the statistically significant differences were also clinically significant.

Multiple linear regression analysis was applied to repolarization markers such as QTc and Tp-e/QTc to evaluate the independent effect of serum magnesium level. The dependent variables were QTc, Tp-e, Tp-e/QT, and Tp-e/QTc. Although QTc mathematically corrects for heart rate, residual heart-rate dependence—particularly when using Bazett’s formula—may persist; therefore, heart rate was retained as a covariate in the regression models.

Independent variables:

- Serum magnesium level.
- Age, gender.
- Potassium, calcium, creatinine.
- Heart rate, QRS duration, BMI.

Variance Inflation Factor (VIF) was examined for multiple linearity in the regression model, and variables with VIF > 5 were excluded from the model.

Correlation analyses were performed to evaluate the relationships between serum magnesium levels and ventricular repolarization parameters. Appropriate correlation tests were selected according to the distribution characteristics of the variables. Pearson correlation analysis was calculated for parameters with normal distribution, and Spearman rho correlation coefficient was calculated for parameters without normal distribution. The obtained correlation coefficients (r) were interpreted based on threshold values ​​widely accepted in the literature to determine the strength of the relationship. Accordingly, an r value between 0.1 and 0.3 was considered weak, between 0.3 and 0.5 was moderate, and above 0.5 was considered strong. These analyses were performed to quantitatively reveal the effects of changes in serum magnesium levels on ventricular repolarization. The study power was calculated in advance using G*Power 3.1.9.7 software. With 80 individuals per group, we aimed to detect at least a moderate effect (Cohen’s d ≥ 0.5) at α = 0.05 and β = 0.20 (power = 80%).

## Results

A total of 160 individuals were included in the study. Participants were divided into two groups according to their serum magnesium levels: hypomagnesemia (*n* = 80; <1.7 mg/dL) and normomagnesemia control group (*n* = 80; 1.8–2.2 mg/dL). No statistically significant difference was found between the groups in terms of age, gender, body mass index (BMI), left ventricular ejection fraction (LVEF), left atrial diameter, potassium, calcium, creatinine, hemoglobin, and other biochemical values ​​(*p* > 0.05). The serum magnesium level was significantly lower in the hypomagnesemia group as expected (1.50 ± 0.12 mg/dL vs. 2.01 ± 0.10 mg/dL; *p* < 0.001) (Table 1).

**Table 1:** Comparison of Clinical, Echocardiographic, and biochemical parameters between hypomagnesemia and control groups

	Hypomagnesemia group (*n* = 80) Mean ± SD	Control group (*n* = 80) Mean ± SD	*P*
Age (years)	53.73 ± 10.12	53.81 ± 10.53	0.957
BMI (kg/m^2^)	26.68 ± 2.75	27.02 ± 3.12	0.466
LVEF (%)	60.13 ± 4.90	60.19 ± 4.32	0.942
LA Diameter (mm)	38.03 ± 3.51	38.42 ± 3.35	0.466
IVS (mm)	10.65 ± 1.14	10.45 ± 1.21	0.277
Hemoglobin (g/dL)	13.67 ± 0.89	13.41 ± 1.06	0.094
Hematocrit (%)	41.39 ± 2.99	41.00 ± 2.55	0.379
Sodium (mmol/L)	137.96 ± 1.93	138.05 ± 2.24	0.792
Potassium (mmol/L)	4.08 ± 0.21	4.05 ± 0.21	0.373
Urea (mg/dL)	32.50 ± 6.17	31.92 ± 7.89	0.611
Creatinine (mg/dL)	1.02 ± 0.18	1.02 ± 0.20	0.940
Calcium (mg/dL)	9.21 ± 0.19	9.21 ± 0.20	0.885
Magnesium (mg/dL)	1.50 ± 0.12	2.01 ± 0.10	**< 0.001**

Abbreviations, *BMI*, Body Mass Index, *LVEF*, Left Ventricular Ejection Fraction, *LA*, Left Atrium, *IVS*, Interventricular Septum

In group comparisons of electrocardiographic parameters, QT (407.80 ± 17.59 ms vs. 394.45 ± 17.81 ms; *p* < 0.001), QTc (431.72 ± 17.82 ms vs. 414.55 ± 18.23 ms; *p* < 0.001) and Tp-e (95.31 ± 10.38 ms vs. 82.88 ± 7.89 ms; *p* < 0.001) intervals were observed to be significantly prolonged in the hypomagnesemia group compared to the control group. Likewise, Tp-e/QT (0.23 ± 0.03 vs. 0.21 ± 0.02; *p* < 0.001) and Tp-e/QTc (0.22 ± 0.03 vs. 0.20 ± 0.02; *p* < 0.001) ratios, which are indicators of repolarization dispersion, were significantly higher in the hypomagnesemia group. QRS duration was significantly longer in the hypomagnesemia group than in the control group (96.77 ± 9.27 ms vs. 93.26 ± 11.50 ms; *p* = 0.035). No significant difference was found between the groups regarding heart rate (77.63 ± 6.77 bpm vs. 76.16 ± 6.74 bpm; *p* = 0.169) (Table 2). Effect size analysis further supported the clinical relevance of these findings, demonstrating large effect sizes for QTc and Tp-e and a moderate effect size for Tp-e/QTc.

**Table 2:** Comparative ECG parameters between groups

	Hypomagnesemia group (*n* = 80) Mean ± SD	Control group (*n* = 80) Mean ± SD	*P*
Heart rate (bpm)	77.63 ± 6.77	76.16 ± 6.74	0.169
Tp-e (ms)	95.31 ± 10.38	82.88 ± 7.89	**< 0.001**
QT (ms)	407.80 ± 17.59	394.45 ± 17.81	**< 0.001**
QTc (ms)	431.72 ± 17.82	414.55 ± 18.23	**< 0.001**
Tp-e/QT	0.23 ± 0.03	0.21 ± 0.02	**< 0.001**
Tp-e/QTc	0.22 ± 0.03	0.20 ± 0.02	**< 0.001**
QRS (ms)	96.77 ± 9.27	93.26 ± 11.50	0.035

Effect size analysis demonstrated large effects for QTc (Cohen’s d = 0.95) and Tp-e (Cohen’s d = 1.36), and a moderate effect for Tp-e/QTc (Cohen’s d = 0.71), supporting the clinical relevance of the observed differences.

In Spearman correlation analyses performed between serum magnesium levels and ventricular repolarization parameters, a significant negative correlation was found between magnesium levels and QTc (*r* = − 0.437, *p* < 0.001), Tp-e (*r* = − 0.457, *p* < 0.001), Tp-e/QT (*r* = − 0.288, *p* = 0.002) and Tp-e/QTc (*r* = − 0.281, *p* = 0.003). These results show ventricular repolarization heterogeneity increases as magnesium levels decrease (Table 3).

**Table 3:** Correlation between magnesium and ventricular repolarization parameters

Variable	Correlation coefficient (*r*)	*p*
QTc (ms)	-0.437	**< 0.001**
Tp-e (ms)	-0.457	**< 0.001**
Tp-e/QT	-0.288	**0.002**
Tp-e/QTc	-0.281	**00003**

All correlations were calculated using the Spearman test and were found to be statistically significant (*p* < 0.05).

**Fig. 2:**
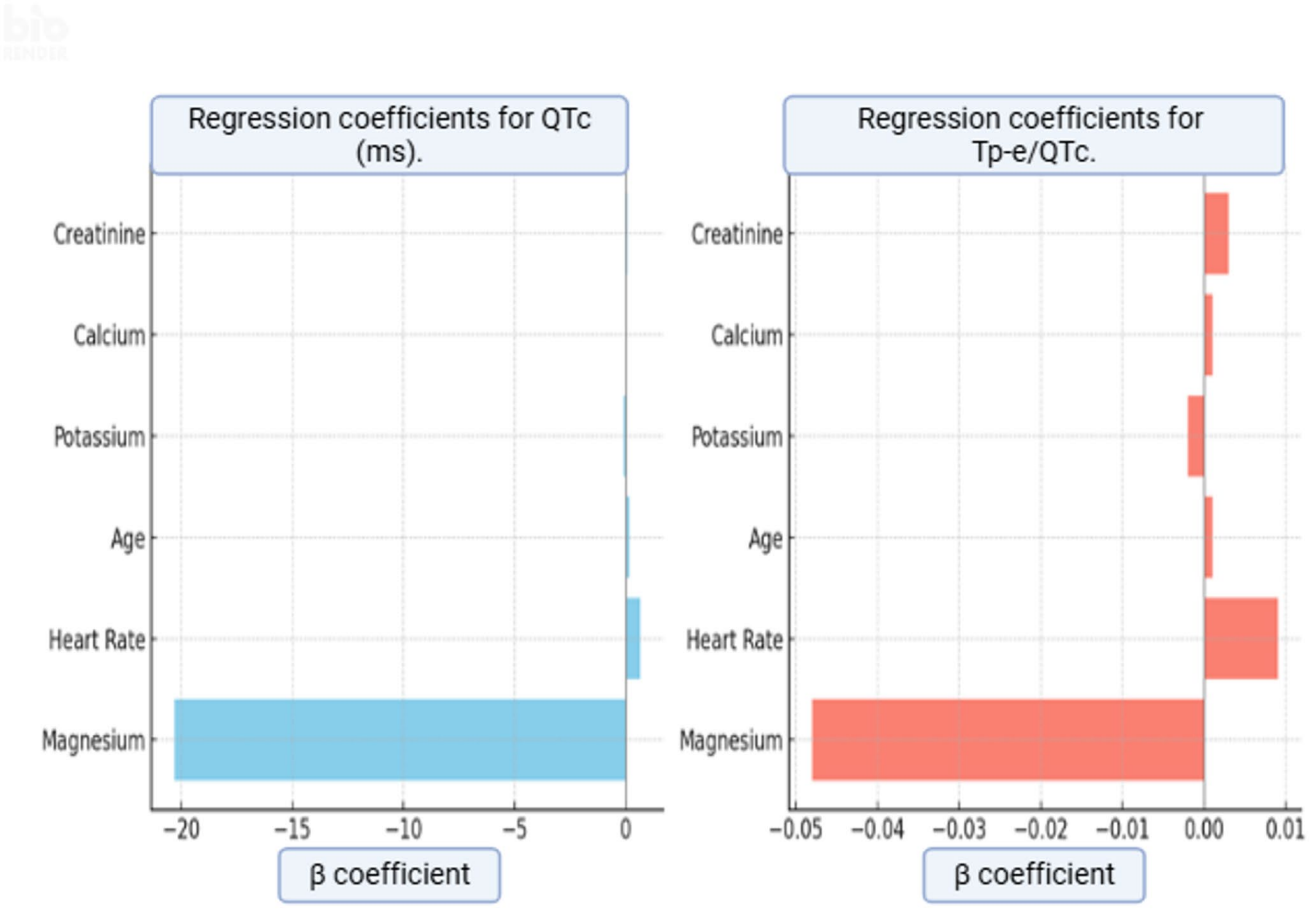
β Coefficients of Variables in Regression Models

Multiple linear regression analyses were performed to determine the independent variables affecting QTc duration and Tp-e/QTc ratio. Importantly, QRS duration was included in the multivariable regression models, and serum magnesium remained independently associated with QTc and Tp-e/QTc, suggesting that the observed repolarization differences were independent of depolarization-related changes. When age, heart rate, potassium, calcium, and creatinine were controlled, serum magnesium level was an independent and significant predictor for both parameters. Table 4. Figure 2.

**Table 4:** Multiple linear regression results for QTc and Tp-e/QTc parameters

Variable	QTc β (*p*-value)	Tp-e/QTc β (*p*-value)	Comment
Magnesium	-20.3 (*p* < 0.001)	-0.048 (*p* = 0.001)	Significant negative predictor
Heart Rate	0.64 (*p* = 0.002)	0.009 (*p* = 0.154)	Significant for QTc
Age	0.15 (*p* = 0.071)	0.001 (*p* = 0.659)	Insignificant
Potassium	-0.08 (*p* = 0.121)	-0.002 (*p* = 0.246)	Insignificant
Calcium	0.02 (*p* = 0.385)	0.001 (*p* = 0.747)	Insignificant
Creatinine	0.05 (*p* = 0.446)	0.003 (*p* = 0.505)	Insignificant
Gender	0.82 (*p* = 0.311)	0.004 (*p* = 0.418)	Insignificant
QRS duration	0.21 (*p* = 0.028)	0.002 (*p* = 0.094)	Insignificant
BMI	0.17 (*p* = 0.192)	0.001 (*p* = 0.512)	Insignificant

**Fig. 3:**
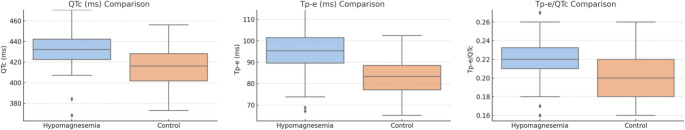
Box plots comparing QTc, Tp-e, and Tp-e/QTc values ​​between hypomagnesemia and control groups. All parameters were elevated in the hypomagnesemia group

Figure 3 present boxplots visually showing group comparisons of QTc, Tp-e, and Tp-e/QTc parameters. These graphs show a significant prolongation of repolarization time in the hypomagnesemia group.

## Discussion

This study found significant relationships between hypomagnesemia and ventricular repolarization parameters. The fact that QT, QTc, Tp-e, Tp-e/QT, and Tp-e/QTc values ​​were significantly prolonged in individuals with low serum magnesium levels compared to the control group demonstrates once again the central role of magnesium in the regulation of cardiac electrical activity at the clinical level. These results provide important clues about the pathophysiological effects of magnesium on the ventricular repolarization process beyond just a biochemical deficiency.

Magnesium ions play a fundamental role in ensuring the membrane stability of the myocardial cell, primarily through sodium-potassium and calcium channels [18, 19]. In this context, it has been shown in experimental and clinical studies that magnesium deficiency prolongs the repolarization period in cardiomyocytes, increases the dispersion of transmural repolarization, and thus paves the way for malignant ventricular arrhythmias such as torsades de pointes [20–22]. In our study, the negative correlation of parameters such as QTc and Tp-e/QTc with magnesium levels and the fact that magnesium stands out as an independent determinant in multiple linear regression models support the fact that these biological mechanisms have significant clinical implications in the human population.

Compared with previous studies, our findings are consistent with the descriptive studies of Gupta et al., indicating that QTc and Tp-e/QT ratios reflect arrhythmogenic potential. Although the observed QTc values remained within conventionally accepted normal limits, the approximately 17 ms intergroup difference may reflect subclinical ventricular repolarization heterogeneity rather than overt QT prolongation. In this context, Tp-e–derived indices, which are considered more sensitive markers of transmural dispersion, may provide additional electrophysiological insight beyond absolute QTc thresholds. In Gupta’s study, Tp-e and Tp-e/QT ratios were reported to increase significantly in events such as torsades de pointes and ventricular fibrillation [23]. Our study showed that these parameters were significantly affected not only in arrhythmia events but also in subclinical electrolyte imbalances such as magnesium deficiency. Similarly, in the studies conducted by Pham et al. in type 2 diabetic individuals, a statistically significant relationship was found between hypomagnesemia and QTc prolongation; however, Tp-e and its derivative parameters were not evaluated [24]. In this respect, our study contributes to the literature, going beyond classical repolarization markers and evaluating more sophisticated electrocardiographic reflections of transmural heterogeneity.

In addition, the regression models obtained in our study showed that heart rate and magnesium levels have a joint effect on QTc. This situation shows that while QTc maintains its physiological dependence on heart rate, magnesium deficiency can directly modify this process. This again emphasizes that electrolyte levels should not be neglected when evaluating QTc in clinical practice.

However, the fact that new generation repolarization parameters such as Tp-e/QTc are significantly affected by magnesium deficiency offers potential contributions to developing noninvasive markers that will allow earlier and more sensitive determination of cardiovascular risk. A comprehensive review of arrhythmia mechanisms by Tse et al. reported that the Tp-e/QTc ratio reflects the dispersion of transmural repolarization and that an increase in this ratio is directly related to arrhythmogenic risk [25].

One of the important contributions of this study to the literature is that it goes beyond the classical limits of ECG parameters and demonstrates the effects of a frequently seen and often overlooked condition, such as magnesium deficiency, on the repolarization process both quantitatively and statistically. In addition, the study’s homogeneous sample, the control of possible confounding variables (such as potassium, calcium, and creatinine), and the use of powerful analysis methods such as multiple linear regression increase its value from a methodological perspective. The single-center and retrospective nature of the study does not allow us to establish a causal relationship due to its observational nature. In addition, there is no follow-up data on the long-term development of arrhythmic events in the individuals evaluated. Future prospective and multicenter studies may reveal the clinical predictive value of these parameters more robustly.

This study demonstrates that serum magnesium level has an independent and significant effect on ventricular repolarization. Hypomagnesemia may increase arrhythmogenic risk by increasing transmural repolarization dispersion. Therefore, carefully assessing magnesium levels, especially in individuals with high cardiac risk, may provide earlier detection of ventricular arrhythmia risk through a simple and accessible biomarker. This study demonstrates that serum magnesium level has an independent and significant effect on ventricular repolarization. Hypomagnesemia may increase arrhythmogenic risk by increasing transmural repolarization dispersion. Therefore, carefully assessing magnesium levels, especially in individuals with high cardiac risk, may provide earlier detection of ventricular arrhythmia risk through a simple and accessible biomarker.

## Conclusion

This study has shown that hypomagnesemia has significant effects on ventricular repolarization parameters and that this effect can be objectively monitored with both classical (QT, QTc) and new generation (Tp-e, Tp-e/QT, Tp-e/QTc) electrocardiographic indicators. The significant negative correlations between serum magnesium levels and ventricular repolarization and the determination of magnesium level as an independent predictor in multiple regression analyses emphasize the critical role of this electrolyte in cardiac electrical stability. The prolongations detected in parameters reflecting arrhythmogenic risk, such as QTc and Tp-e/QTc, especially suggest that ventricular repolarization heterogeneity increases in the presence of hypomagnesemia and may have clinical significance. In line with the obtained data, it has been concluded that early and regular evaluation of magnesium levels can be used as an important biomarker in preventing arrhythmic complications. Accordingly, evaluating magnesium levels in individuals at risk for ventricular arrhythmia can be integrated into clinical practice as a low-cost, widely accessible, and effective preventive approach. Confirming these findings more strongly with future large-scale, prospective studies is of great importance.

## Data Availability

The data supporting the findings of this study are available from the corresponding author upon reasonable request and subject to institutional regulations.
